# Treatment challenges in glioblastoma: a case report

**DOI:** 10.3389/fonc.2025.1651939

**Published:** 2026-01-07

**Authors:** Domingos Roda, Filipa Oliveira, Pedro Veiga, Leonor Santos, Pedro Abreu, Ana Nascimento, Francisco Caramelo, Joana Barbosa Melo, Isabel Marques Carreira, Ilda Patrícia Ribeiro

**Affiliations:** 1 Algarve Radiation Oncology Unit, Joaquim Chaves Saúde (JCS), Faro, Portugal; 2 Cytogenetics and Genomics Laboratory, Institute of Cellular and Molecular Biology, Faculty of Medicine, University of Coimbra, Coimbra, Portugal; 3 Radiation Oncology Service, Unidade Local de Saúde de Coimbra, Coimbra, Portugal; 4 Neurosurgery Service, Unidade Local de Saúde do Algarve, Algarve, Portugal; 5 Laboratory of Biostatistics and Medical Informatics, Instituto Biomédico de Investigação em Luz e Imagem (IBILI) - Faculty of Medicine, University of Coimbra, Coimbra, Portugal; 6 Coimbra Institute for Clinical and Biomedical Research (iCBR) and Centre of Investigation on Environment Genetics and Oncobiology (CIMAGO), Faculty of Medicine, University of Coimbra, Coimbra, Portugal; 7 Center for Innovative Biomedicine and Biotechnology (CIBB) and Clinical Academic Centre of Coimbra (CACC), University of Coimbra, Coimbra, Portugal

**Keywords:** glioblastoma, liquid biopsies, circulating tumour DNA, genetic and epigenetic signature, copy number alterations

## Abstract

This clinical case report aimed to show the survival timeline of a glioblastoma patient who underwent surgery and received adjuvant treatments in accordance with the Stupp protocol. Starting on the day of surgery, a liquid biopsy was employed to assess the dynamic alterations in cell-free DNA (cfDNA). Upon recurrence, reoperation was conducted, and repeated radiochemotherapy was applied. This clinical case is interesting due to the extended survival time, despite the extremely aggressive nature of the medical interventions. Liquid biopsies constitute a non-invasive method of characterising circulating tumour DNA (ctDNA) and evaluating genetic and epigenetic modifications, providing considerable potential for tumour diagnosis, prognosis, and management.

## 1 Introduction

Glioblastoma (GB), accounting for approximately 50% of all primary central nervous system (CNS) neoplasms, is the predominant primary malignant brain tumour in adults, with an annual incidence of roughly three cases per 100,000 individuals worldwide (1). The surgical intervention, if viable, is the primary therapy modality for individuals with newly diagnosed GB. Nonetheless, GB is an extensively diffusive, invasive, and vascularised neoplasm and is not amenable to surgical cure. Consequently, concurrent and adjuvant temozolomide (TMZ), in combination with radiation therapy, has been considered the standard treatment following surgery (2). Notwithstanding these intensive therapies, the median overall survival of GB since diagnosis in treated patients is approximately 14 months (3). Treatment outcomes have mostly remained static in recent decades, with the majority of GB patients experiencing tumour recurrence. The mean progression-free survival (PFS) is approximately 7 months since diagnosis (2). Recurrence of GB has been unavoidable, and nearly all patients will experience relapse, predominantly occurring centrally within 2 cm of the first gadolinium-enhanced mass on magnetic resonance imaging (MRI) (4). In this circumstance, there is no established treatment. Surgical re-intervention rates rarely go above 30%, with certain studies indicating that a second surgery can be conducted in fewer than 10% of individuals (5). Various therapeutic alternatives can be considered, including reoperation, reirradiation, or a combination of both. Chemotherapy (CT), especially nitrosoureas, and antiangiogenic agents, such as bevacizumab, may be used alone or in combination with radiotherapy.

We present herein a case report of an operated patient who, according to the Stupp protocol, underwent radiochemotherapy followed by chemotherapy in monotherapy. From the day of surgery, liquid biopsy was used to evaluate the dynamic changes in cell-free DNA (cfDNA), and molecular characterisation of tumour tissue was also performed. When recurrence occurred, reoperation was performed, followed by repeated radiochemotherapy, with the addition of bevacizumab. The uniqueness of this clinical case is related to the extended survival time, maintaining, despite very aggressive medical interventions, an excellent general condition that allows him to maintain his working life as a construction worker.

## 2 Clinical case

**Figure 1:**
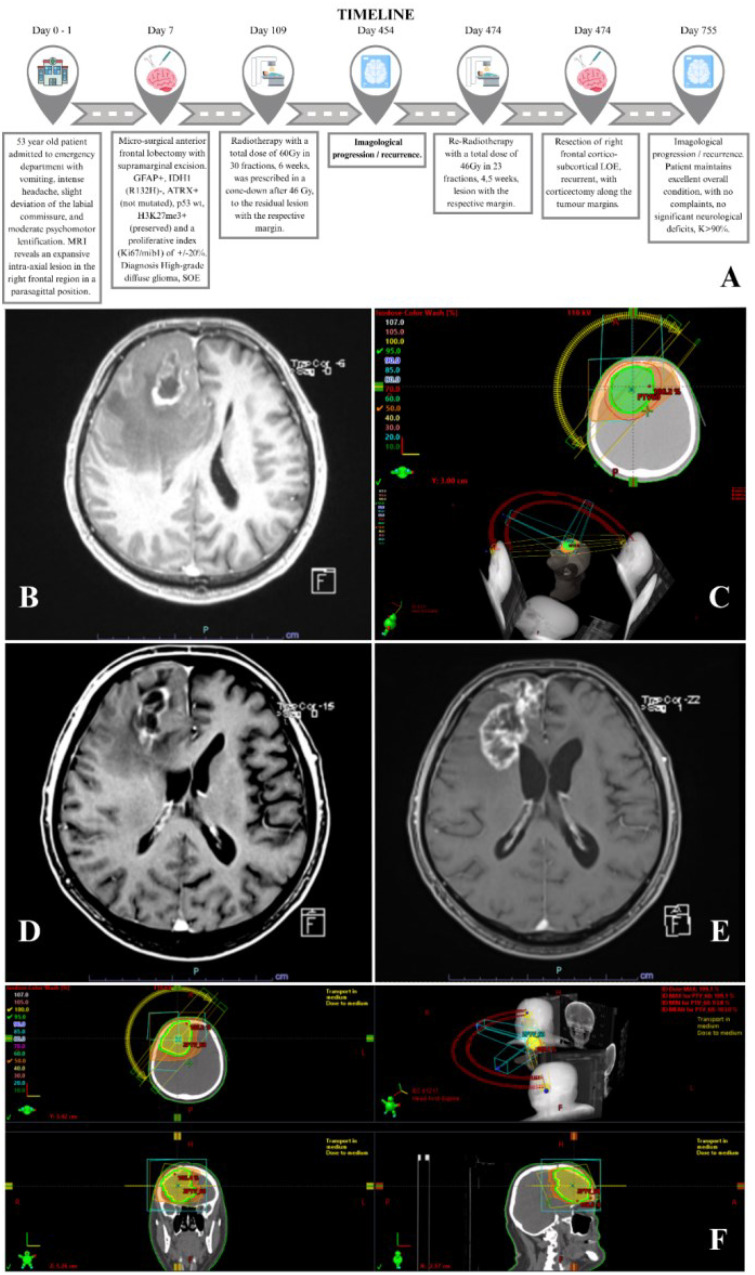
**(A)** Case report timeline. **(B)** Diagnostic MRI. **(C)** Radiotherapy isodose lines/beam configuration (initial treatment). **(D)** MRI showing pseudo-progression/necrosis. **(E)** MRI showing progression. **(F)** Radiotherapy isodose lines/beam configuration (re-irradiation).

A 53-year-old Ukrainian patient residing in Portugal with family, with no significant past medical history and who was not on any chronic medication, was admitted to the emergency department on March 16, 2023 (day 0) (**Figure 1A**). The patient presented with a 7-day history of gradual evolution of symptoms, characterised by vomiting, intense headache, slight deviation of the labial commissure, and moderate psychomotor lentification. Following clinical evaluation, complementary diagnostic tests were performed, with imaging tests being particularly highlighted.

The MRI, performed on March 17, 2023 (day 1), revealed an expansive intra-axial lesion in the right frontal region in a parasagittal position, characterised by an apparently cystic/necrotic component in the right parasagittal frontal area associated with extensive perilesional vasogenic oedema. After contrast injection, heterogeneous enhancement was observed at the margins of this cystic/necrotic lesion, which had an Anteroposterior (AP) diameter of 43 mm, a transverse diameter of 24 mm, and a cranio-caudal diameter of 43 mm. The perilesional vasogenic oedema was minimal (**Figure 1B**).

While the patient was still in the emergency department, anti-oedematous therapy with dexamethasone was initiated.

This patient gave consent to be enrolled in a scientific project focused on the genomic characterisation of GB tumour samples and cfDNA quantification. The study protocol includes the collection of peripheral blood immediately prior to the neurosurgical procedure, fresh processing of the surgical specimen, and sequential blood sampling during chemoradiotherapy and throughout follow-up. This study was approved by the Committee on Ethics in Research of the Faculty of Medicine of the University of Coimbra, and written informed consent was obtained from the patient. All the experiments were performed according to the regulations in the Declaration of Helsinki.

The patient was electively admitted to the neurosurgery department and, after peripheral blood collection, underwent a micro-surgical anterior frontal lobectomy with supramarginal excision on March 24, 2023 (day 7), respecting the branches of the callosomarginal artery at the posterior tumour margin, guided by neuronavigation. The anatomic-pathological study described an infiltrative glial neoplasm with slight cellular pleomorphism, microvascular proliferation, and areas of necrosis. Microcystic areas and some scattered calcospherites were observed. There was an accumulation of cells in the subpial region and perivascular invasion. The immunohistochemical study revealed the following: GFAP+, IDH1 (R132H)−, ATRX+ (not mutated), p53 wt, H3K27me3+ (preserved), and a proliferative index (Ki67/mib1) of ±20%. Diagnosis of high-grade diffuse glioma was made. After DNA extraction, PCR amplification, and bidirectional sequencing using the Sanger method, no mutational variants were detected at codon 132 (Arg132) of the *IDH1* gene or at codon 172 (Arg172) of the *IDH2* gene.

The complete recovery of neurological deficits was evident, having progressed favourably during the hospitalisation, with discharge after 6 days of the surgery.

The clinical situation was discussed in a therapeutic multidisciplinary meeting 3 days after the surgery, and chemoradiotherapy was proposed according to the standardised “Stupp protocol”.

The patient was evaluated in a radiotherapy consultation on May 10, 2023, having undergone the immobilisation mask, the planning CT, and the planning MRI on the same day. A total dose of 60 Gy in 30 fractions, during 6 weeks, was prescribed in a cone-down after 46 Gy to the residual lesion with the respective margin, with 6- and 15-MV photons, using the Intensity modulated radiation therapy– volumetric modulated arc therapy (IMRT–VMAT) technique, through daily image guidance on TrueBeam^®^.

A volumetric brain MRI was performed with and without contrast for planning. Gross tumour volume 1 (GTV 1) encompassed the resection cavity, residual enhancement on postoperative T1 postcontrast, and T2/Fluid Attenuated Inversion Recovery (FLAIR) changes. GTV 2 was equal to GTV 1 without the T2/FLAIR changes (non-enhancing tumour). Clinical target volumes (CTVs) 1 and 2 were equal to GTV 1/2 with a 15-mm expansion, modified to respect natural anatomical barriers. Planning target volume (PTV) 1/2 resulted from a 2-mm geometrical expansion of CTV 1/2.

Simultaneously, the patient started TMZ at a dose of 75 mg/m^2^ per day, with daily intake.

The radiotherapy phase began on May 22, 2023 (day 67) and ended on July 3, 2023. The delineated volumes and the respective dose distribution curves are depicted in **Figure 1C**. This was followed by adjuvant TMZ (d1–5) for a 28-day cycle for 6 months, starting at a dose of 150 mg/m^2^ and gradually escalating to 200 mg/m^2^.

The patient maintained regular blood sampling during the maintenance of the treatment phases. A gradual and progressive tapering of dexamethasone was carried out, which was completed on July 31, 2023.

On November 27, 2023, during the oncology consultation, a neurological worsening characterised by left hemiparesis grade 4 was detected, coinciding with the fifth cycle of temozolomide. At this stage, dexamethasone (4 mg) was started, with the patient showing significant improvement and complete reversal, and was totally withdrawn on January 15, 2024. The sixth and last cycle of TMZ was administered on December 22, 2023.

Multiple imaging studies were conducted with suspicion of pseudo-progression/necrosis, with the comparative MRI on February 12, 2024 (day 333), demonstrating changes in the morphology of the lesion, with low signal in Susceptibility-Weighted Imaging (SWI), now characterised by increased thickness and irregularity, with more evident enhancement involving the subcortical and periventricular/frontal horn adjacent regions as well as enhancement of the dura adjacent to the lesion, suggesting possible progression/pseudo-progression of the disease (**Figure 1D**). At this stage, there were no motor deficits, only moderate dysarthria.

It was decided to maintain tight imaging and clinical surveillance, as the patient maintained a Karnofsky Performance Status (KPS) of 100%, continuing to work as always as a construction worker. New clinical deterioration was detected in May 2024 with the resurgence of left hemiparesis, G3.

The MRI performed on June 12, 2024 (day 454), described an increase in the dimensions of the lesion occupying the right frontal cortico-subcortical space, extending to the surface of the frontal extension of the right lateral ventricle, measuring approximately 60 × 30 × 45 mm (AP × LL × CC). It presented a heterogeneous signal in T1 and T2, with thick peripheral signal enhancement, centrally necrotic–cystic. In the perfusion study, an increase was observed at the solid margins of the lesion. The features were suggestive of a primary neoplastic lesion of the central nervous system (e.g., grade 4 astrocytoma) in progression (**Figure 1E**).

Re-evaluated in a therapeutic decision consultation, he was again proposed for neurosurgical re-intervention because of progressive worsening of left-sided paresis and dysarthria. He was subjected on July 2, 2024 (day 474), to resection of right frontal cortico-subcortical Space Occupying Lesion (SOL) recurrent, with corticectomy along the tumour margins defined by neuronavigation. Histological result confirmed diffuse glioma with high-grade morphological criteria. Analysis of the methylation status of the promoter region of the *MGMT* gene detected a heterogeneous methylation pattern, in which five of the eight probes tested showed methylation. The overall mean methylation percentage detected was 33% [methylation-specific multiplex ligation-dependent probe amplification (MS-MLPA)] (ME012-B1 Probemix, MRC-Holland, Amsterdam, The Netherlands). The C228T mutation in the *TERT* gene promoter was also detected using the probe present in the MS-MLPA Probemix used. The amplification of the *EGFR* gene was identified.

The patient had functional recovery after surgery. He was proposed for a repetition of the “Stupp protocol - chemoradiation”, considering his age, excellent general condition, and pathological corroboration. He resumed chemoradiotherapy on August 19, 2024 (day 522) with a total dose of 46 Gy/23 fractions, for 4.5 weeks, in the context of re-irradiation, which concluded on September 18, 2024, with excellent tolerance (**Figure 1F**). The technique was performed on a one-phase approach with conventionally fractionated radiotherapy (RT). GTV included residual contrast-enhancing tumour identified on postcontrast T1-weighted MRI images, non-enhancing tumour, and resection cavity. GTV expansion of 3 mm to CTV was performed, cropping natural barriers. CTV expansion of 2 mm to PTV was performed. Cumulative doses were below standard dose constraints. During the RT course, the TMZ dose was 75 mg/m^2^. In monotherapy, it was escalated, according to the haematological profile, to 150 mg/m^2^. It was finalised on April 1, 2025.

The most recent MRI performed on March 24, 2025 (day 739), described the progression of the disease through imaging: a significant increase in the necrotic lesion on the posterior aspect adjacent to the surgical site with enhancement and areas of restriction measuring 7.7 × 5.3 × 6 cm (AP × LL × CC, previous exam 5.3 × 4.9 × 5.5 cm). New contrast uptake in the knee of the corpus callosum was observed.

In the last oncology consultation on April 9, 2025 (day 755), it was reported that the patient maintained excellent overall condition, with no complaints, no significant neurological deficits, and KPS > 90%. Presenting imaging progression on MRI that precedes the still non-evident clinical progression. After multidisciplinary discussion, it was proposed to start lomustine at a dose of 90 mg/m^2^ every 6 weeks (maximum dose, 160 mg) plus bevacizumab at a dose of 10 mg per kilogram of body weight every 2 weeks, according to the BELOB trial.

### 2.1 Molecular findings

#### 2.1.1 Tumour tissue characterisation

DNA extraction from tumour tissue was performed using High Pure PCR Template Preparation Kit (Roche GmbH, Mannheim, Germany), according to the manufacturer’s instructions. In tumour tissue, copy number alterations (CNAs) were analysed through array comparative genomic hybridisation (aCGH) using Agilent SurePrint G3 Human Genome microarray 180K (Agilent Technologies, Santa Clara, CA, USA) as we previously described (6). MS-MLPA using the ME002 SALSA Probemix (MRC-Holland, Amsterdam, The Netherlands) was also performed in order to simultaneously evaluate the CNAs and methylation patterns in a specific set of genes, as we previously described (7).

**Figure 2:**
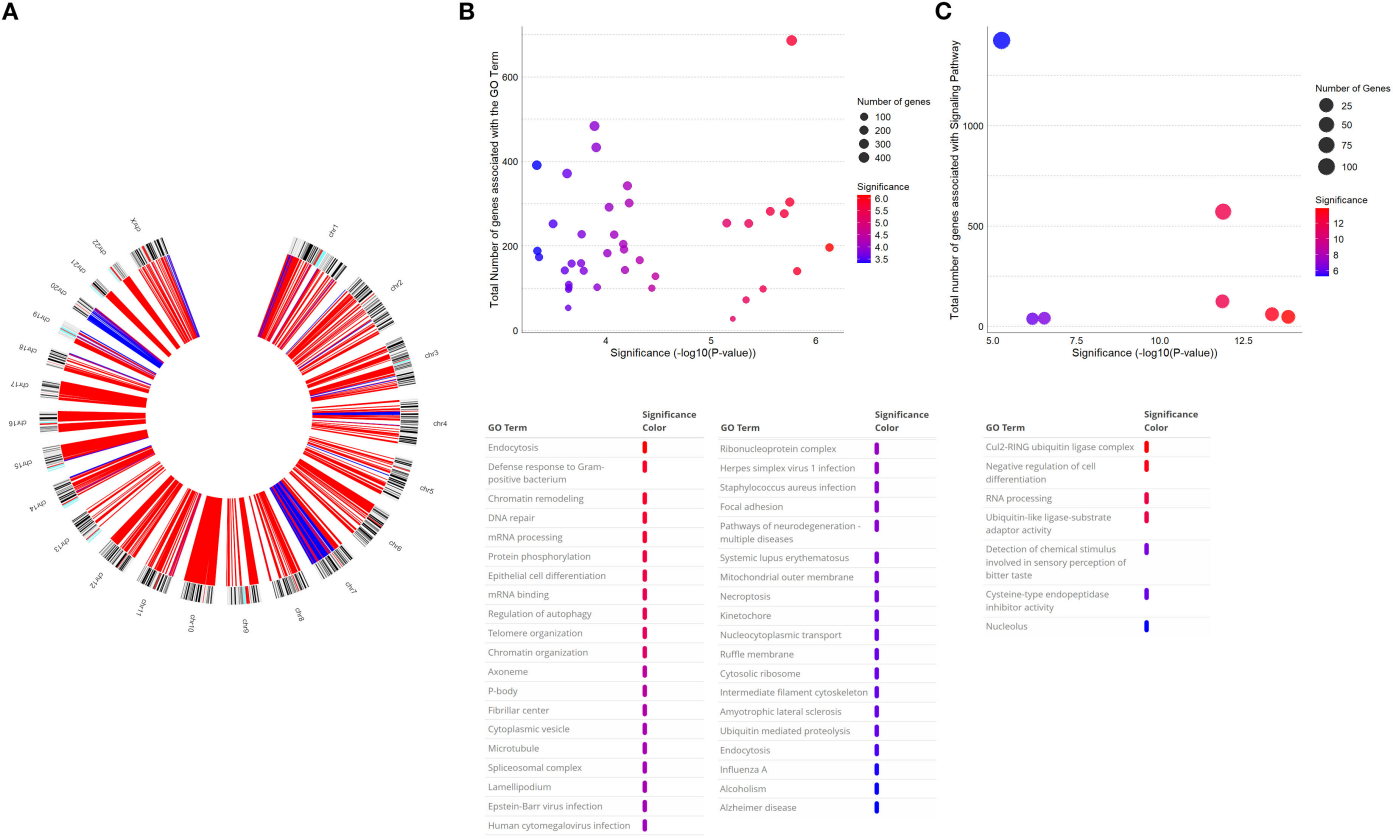
**(A)** Circus plot with aberration pattern of GB tumour tissue detected using aCGH technique. Blue represents copy number gains, and red represents copy number losses. **(B, C)** Bubble plot of significantly enriched [p < 0.01 **(B)** and p < 0.05 **(C)**] GO terms associated with deleted **(B)** and amplified **(C)** genes in the aCGH analysis of this patient. The x-axis represents the −log10(p-value), and the y-axis the total number of genes involved in a particular signalling pathway. The size of the circles represents the number of genes amplified and deleted in the patient. At the bottom, the GO terms present in the chart are associated with the significance. GB, glioblastoma; aCGH, comparative genomic hybridisation.

Whole-genome approach, aCGH, revealed several chromosomal abnormalities, with predominance of chromosomal losses (**Figure 2A**). Copy number deletions in entire chromosomes were observed, namely, at chromosomes 10, 16, 17, 21, and 22, while no chromosomes were completely amplified. Additionally, several chromosomes exhibited partial copy number losses or gains. Overall, deletions were more frequent, presenting in 11 chromosomes, while amplifications were identified in only three, as described in **Table 1**.

**Table 1:** Copy number gains and losses identified in this patient’s GB tumour tissue via aCGH, including chromosomal location, mapped gene functions, alteration size, and supporting literature on their roles in cancer.

Chromosome	Selected genes (location)	Type of alteration	Role	Chromosomal alteration size	References
1	*ARID1A* (1p36.11)	Deletion	Therapy response	38.0 Mb	(16)
	*CDKN2C* (1p32.3)	Deletion	Therapy response		(14)
4	*KIT* (4q12)	Amplification	Signalling pathways	11.9 Mb	(8)
	*PDGFRA* (4q12)	Amplification	Signalling pathways		(17)
6	*TAP1* (6p21.32)	Deletion	Immunotherapy response	41.4 Mb	(18)
7	*EGFR* (7p11.2)	Amplification	Signalling pathways	1.8 Mb	(8)
	*MET* (7q31.2)	Amplification	Signalling pathways	81.5 Mb	(8)
	*CDK6* (7q21.2)	Amplification	Proliferation and invasiveness		(19)
	*ABCB1* (7q21.12)	Amplification	Proliferation and invasiveness		(20)
	*TES* (7q31.2)	Amplification	Proliferation and invasiveness		(21)
	*BRAF* (7q34)	Amplification	Proliferation and invasiveness		(9)
	*FOXP2* (7q31.1)	Amplification	Prognosis and survival		(22)
	*CFTR* (7q31.2)	Amplification	Cell proliferation		(23)
9	*CDKN2A* (9p21.3)	Deletion	Therapeutic response	29.4 Mb	(10)
	*ELAVL2* (9p21.3)	Deletion	Prognosis and survival		(24)
	*MTAP* (9p21.3)	Deletion	Prognosis and survival		(25)
	*PTPRD* (9p23)	Deletion	Prognosis and survival		(26)
	*MLLT3* (9p21.3)	Deletion	Prognosis and survival		(27)
10	*PTEN* (10q23.31)	Deletion	Tumour suppressor	92.8 Mb	(11)
	*MGMT* (10q26.3)	Deletion	DNA repair		(12)
	*FGFR2* (10q26.13)	Deletion	Signalling pathways		(13)
12	*APAF1* (12q23.1)	Deletion	Apoptosis	32.3 Mb	(28)
	*CDK4* (12q14.1)	Deletion	Therapeutic response	9.9 Mb	(19)
	*PTPN11* (12q24.13)	Deletion	Prognosis and survival	32.3 Mb	(29)
15	*TP53BP1* (15q15.3)	Deletion	Prognosis	54.1 Mb	(30)
	*ACTC1* (15q14)	Deletion	Invasiveness	1.3 Mb	(31)
	*RAD51* (15q15.1)	Deletion	TMZ response	54.1 Mb	(32)
16	*CDH1* (16q22.1)	Deletion	Invasiveness	43.6 Mb	(33)
17	*TP53* (17p13.1)	Deletion	Tumour suppressor	10.0 Mb	(14)
	*NF1* (17q11.2)	Deletion	Tumour suppressor	55.5 Mb	(15)
20	*AURKA* (20q13.2).	Amplification	Cell-cycle regulator	10.4 Mb	(34)
21	*RUNX1* (21q22.12)	Deletion	Tumour angiogenesis	32.5 Mb	(35)
22	*NF2* (22q12.2)	Deletion	Tumour suppressor	29.8 Mb	(36)

GB, glioblastoma; aCGH, comparative genomic hybridisation.

We selected the genes listed in **Table 1** based on two criteria. First, we considered genes that are most frequently altered in GB, as identified in previous studies (8–15). Second, we analysed chromosomal regions altered by aCGH that exhibited genes already associated with carcinogenesis in several tumours (8, 14, 16–36). Within these chromosomal regions, we highlighted genes whose altered copy numbers could contribute critically to tumour development and its subsequent progression.

These CNAs detected using aCGH were corroborated using the MS-MLPA technique, which also presented copy number gains in *CDK6* and *CFTR*, and losses in *CDKN2A*, *PTEN*, *MGMT*, and *TP53*.

With the Gene Ontology term filtering performed using the DAVID tool, selecting the GOTERM_BP, GOTERM_CC, GOTERM_MF, and KEGG_PATHWAY options, we found 902 GO terms associated with the deleted genes and 296 associated with the amplified genes. These GO terms are related to cellular components, molecular functions, and biological processes. Given the large number of results, we selected the GO terms with a p-value below 0.01 and a Benjamini value below 0.01 in the deletions, and GO terms with a p-value below 0.05 and a Benjamini value below 0.02 in the amplifications, as shown in **Figures 2B and C**. The most significantly enriched GO terms associated with the amplified genes were seven, while 39 were associated with the deleted genes. The most enriched terms are related to cell proliferation, DNA damage response and repair, chromatin remodelling and organisation, binding processes, and pathways involved in brain diseases and cancer.

Considering the GO terms presented, we highlighted the ones associated with carcinogenesis, namely, chromatin remodelling and organisation, focal adhesion, DNA repair, necroptosis, and telomere organisation, with 416, 167, 260, 198, 110, and 26 associated genes associated, respectively. We also found genes associated with key biological pathways. Specifically, 303 genes were linked to neurodegenerative disease pathways. Notably, this pathway association was observed in the deleted regions, with no significant enrichment detected in the amplified regions.

The MS-MLPA ME002 analysis revealed promoter methylation in the *ESR1*, *WT1*, and *GATA5* genes. A methylation dosage ratio ≥0.25 was considered indicative of promoter hypermethylation. In contrast, we also observed copy number gains in two of these methylated genes, *ESR1* and *GATA5*.

#### 2.1.2 Liquid biopsy

The isolation of cfDNA from plasma was performed using the QIAamp Circulating Nucleic Acid Kit (Qiagen, Hilden, Germany) according to the manufacturer’s instructions. Total cfDNA quantification was performed using Invitrogen Qubit dsDNA HS Assay Kit and Qubit 3.0 Fluorometer (Life Technologies, Carlsbad, CA, USA) according to the manufacturer’s instructions.

The baseline (pre-treatment) level of cfDNA from this patient was compared with that of controls. The plasma cfDNA concentration of this patient was 0.513 ng/μL at baseline, and that of the controls ranged from 0.006 to 0.793 ng/μL, with a mean concentration of 0.185 ± 0.221 ng/μL. The controls were collected from healthy individuals without a cancer history, with ages between 50 and 80 years.

**Figure 3:**
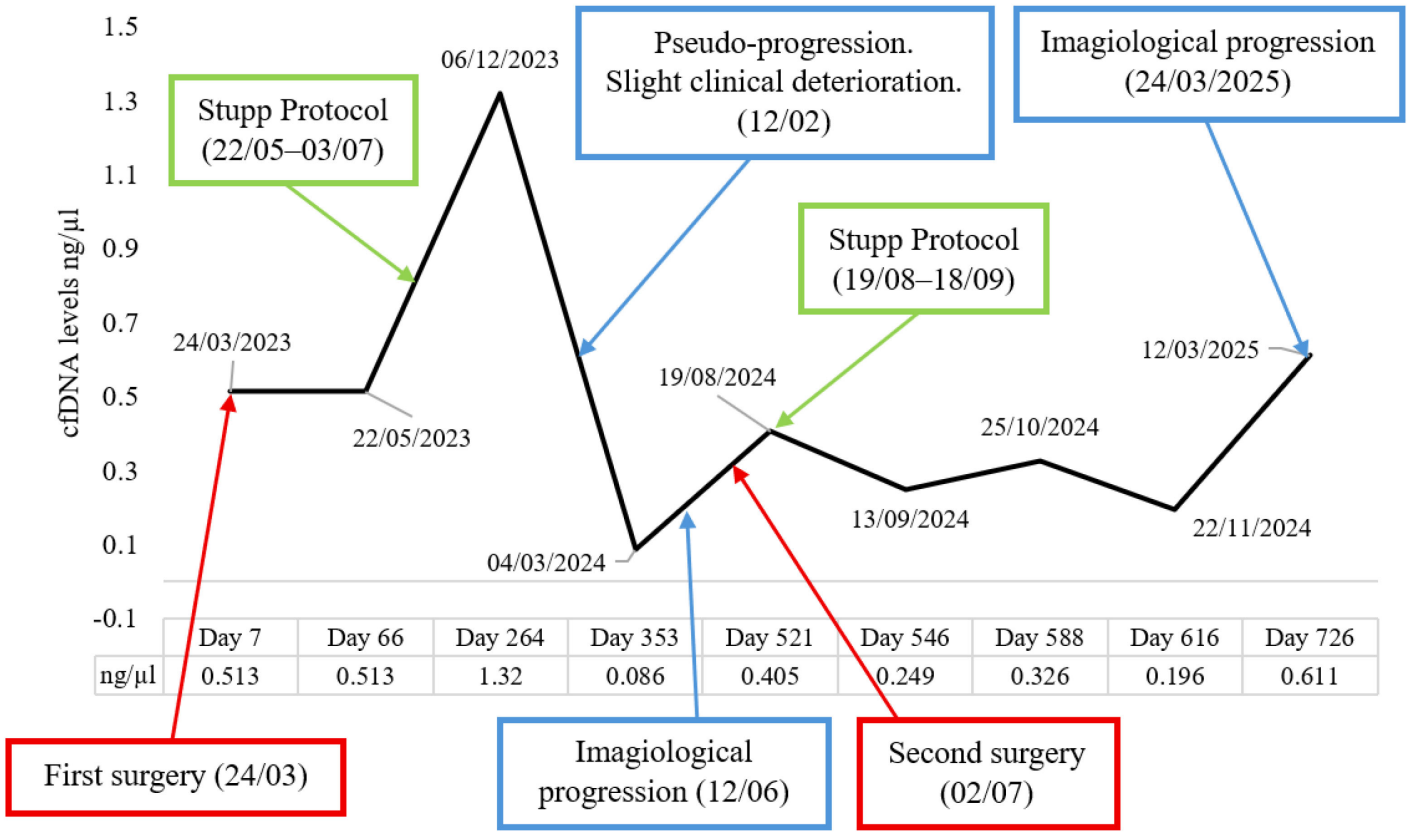
cfDNA concentration in plasma before surgery and at several time points following treatment and progression. The x-axis is not to scale, as the time intervals between sample collections were irregular. cfDNA, cell-free DNA.

To monitor cfDNA levels throughout treatment, nine serial blood samples were collected from this patient: one prior to treatment and eight at various time points following the first surgery and the initiation of therapy. cfDNA was successfully extracted and quantified from all samples at every time point (**Figure 3**).

Plasma cfDNA levels were monitored over the course of treatment. An initial increase in cfDNA concentration was observed following the start of chemoradiotherapy. Approximately 6 months after chemoradiotherapy (QT-RT) initiation, cfDNA levels progressively declined, suggesting a potential therapeutic response. Subsequently, an increase in cfDNA concentration was observed in association with imaging-confirmed tumour progression and prior to the second surgical intervention; however, cfDNA levels did not reach the initial baseline concentrations (possible minimal residual disease). It is important to note that no blood samples were collected at the exact moment of radiological progression or immediately before the second surgery. At the final follow-up time point, cfDNA levels exceeded baseline values, potentially indicating further disease progression.

## 3 Discussion

The genomic analysis of the tumour tissue of this GB patient revealed several chromosomal deletions commonly described in GB samples, such as losses of chromosomes 10, 16, 17, 21, and 22. Key tumour suppressor genes were mapped in these chromosomes, including *TP53* and *NF1* (chr17); *PTEN*, *MGMT*, and *FGFR2* (chr10); *NF2* (chr22); *CDH1* (chr16); and *RUNX1* (chr21). These genes play critical roles in cell cycle regulation, DNA repair, apoptosis, invasiveness, and signal transduction, and their loss has established associations with tumour progression, treatment resistance, and poor prognosis in GB. *TP53*, a tumour suppressor gene, is linked to various cellular processes, such as cellular cycle regulation, programmed cellular death, cellular differentiation, and DNA damage response, frequently found in younger patients and is associated with a better prognosis and overall survival (OS) (11, 37). The *NF1* gene, altered in approximately 20% of GBs, is a regulator of Ras activity in the RAS/MAPK signalling pathway associated with the development of tumours of the central nervous system and a short OS and prognosis (37). *PTEN* has its tumour suppressor function frequently suppressed; this gene is a negative P13K/AKT pathway regulator, frequently associated with mTOR inhibitor sensitivity (9, 37). *MGMT* is an important biomarker of prognosis in GB. This gene codifies a critical protein involved in DNA repair and, when methylated, confers a better response to TMZ and consequently a better prognosis and OS (12). The *FGFR2* gene is involved in the activation of the RAS–MAPK and PI3K–AKT pathways (13), and the *NF2* tumour suppressor gene is typically mutated in most nervous system tumours (36). *RUNX1* is an important regulator of tumour angiogenesis and invasion mediated through the upregulation of key molecules such as matrix metalloproteinases (MMPs) and vascular endothelial growth factor A (VEGFA) (35).

Additional focal deletions, including *CDKN2A*, *CDK4*, *APAF1*, *ARID1A*, and *TP53BP1* genes, which are already implicated in therapeutic response and disease outcome, were also observed in the reported GB patient (10, 16, 19, 30, 38). Copy number losses of the *ELAVL2*, *MTAP*, *PTPRD*, and *MLLT3* genes were associated with prognosis and survival rate in GB (24–27). Deletions of *ARID1A*, which frequently co-occur with *TP53*, and *CDKN2C* are associated with therapy resistance; conversely, *TP53BP1* is associated with a worse OS and poor prognosis (14, 16, 30, 38). Losses of *ACTC1* are associated with invasiveness in GB (31). The deletion of *PTPN11* is also associated with a poor prognosis and low overall survival (29), and the deletion of *RAD51* promotes a better response to TMZ thanks to its central role in homologous recombination-mediated DNA repair, facilitating the repair of double-strand breaks induced by radiotherapy and alkylating agents such as TMZ (32, 39). Recent studies have highlighted the clinical relevance of this gene in GB. Morrison et al. (2021) demonstrated that high *RAD51* expression is significantly associated with reduced overall survival. In this context, the *RAD51* deletion observed in our patient may contribute to a more favourable response to TMZ (40). Overall, the most deleted genes are involved in biological processes associated with tumour growth, protein transport, signal transduction and transcription regulation, cell cycle, apoptotic process, and DNA repair.

The genomic analysis of this patient also revealed chromosome gains, critically involved in tumorigenesis and progression. The amplification of chromosomal regions containing the genes *EGFR*, *MET*, *KIT*, and *PDGFRA* is associated with the receptor tyrosine kinase (RTK) and growth-factor receptors, often driving proliferative and angiogenic signalling in tumours (8, 17). The genes *CDK6*, *ABCB1*, *TES*, and *BRAF* were also amplified in the reported patient; these genes are associated with oncogenic drivers in GB, the MAPK signalling pathway, and the cellular cycle, contributing to uncontrolled proliferation and invasiveness (19–21). The *CFTR* amplification, also found in this patient, is associated with reduced cell proliferation and a better OS and prognosis (23). Also associated with a poor prognosis and worse OS, we found the gene *FOXP2* amplified (22). Altogether, these alterations affect key oncogenic pathways (e.g., PI3K/AKT and RAS/MAPK) and biological processes such as chromatin remodelling, DNA damage response, and immune evasion, reinforcing the aggressive nature and therapeutic challenges of GB.

Upon comparing the methylated genes *GATA5*, *WT1*, and *ESR1* with the aCGH results, we observed that *GATA5* is located within an amplified region, whereas *WT1* is situated in a deleted region; also, *GATA5*, *WT1*, and *ESR1* methylation were associated with poorer outcomes (41).

Despite the coexistence of several genomic alterations generally associated with poor prognosis, including losses of *PTEN*, *TP53*, and *NF1* and amplifications of *EGFR*, *MET*, and *PDGFRA*, this patient continued to display an unexpectedly favourable clinical evolution. Several hypotheses may explain this apparent discrepancy. First, the loss and partial methylation of the *MGMT* promoter (33% overall methylation in five of eight MS-MLPA probes) and the *RAD51* deletion detected may have conferred a degree of sensitivity to temozolomide, contributing to the prolonged disease control observed. Second, the amplification of *CFTR*, which has been associated with decreased tumour proliferation and better survival in gliomas, may also have exerted a protective influence.

Overall, the molecular profile of the described patient reveals several genomic and epigenetic alterations typically associated with poorer overall survival and prognosis. However, in contrast to these expectations, the patient continued to exhibit an excellent clinical condition. This discrepancy underscores the complexity of GB and suggests that additional factors may contribute to individual patient outcomes.

The use of liquid biopsies provides a non-invasive tool for detecting circulating tumour DNA (ctDNA) and assessing genetic and epigenetic alterations, with significant potential to improve tumour diagnosis, prognosis, and management (42, 43). In GB patients, cfDNA levels vary widely, reflecting differences in disease stage, tumour heterogeneity, treatment response, and individual characteristics of each patient (44).

Regarding cfDNA levels in this patient, we see an increase in the levels before the imaging progression, suggesting a possible very early indicator of the disease progression. Interestingly, a gradual reduction was also observed during imaging pseudo-progression, which could be an extremely important finding in this differential diagnosis, especially in an era when robust imaging or nuclear medicine techniques that could eventually allow the differentiation of pseudo-progression from true progression are still scarce, a fundamental issue in clinical decision-making. We can also see the response to treatment when the levels start to decrease, suggesting a favourable response, providing the clinician with a snapshot of the sensitivity or resistance profile to ongoing treatments.

However, it is important to acknowledge the limitations of this study, as it is based on a single patient. The hypotheses generated should therefore be interpreted with caution and further validated in larger, independent cohorts. In addition to quantitative cfDNA analysis, further assessment of tumour-specific genetic alterations should be performed, for example, using Next Generation Sequencing technology, to explore a potential cfDNA molecular signature capable of anticipating disease progression.

## 4 Conclusions

The exhaustive analysis of this clinical case describes the journey of a relatively young patient, who, despite multiple medical and surgical approaches and the aggressiveness of the disease, maintained an acceptable KPS of 25 months after the diagnosis of GB.

This clinical case revealed an extensive chromosomal instability, typical in GB, with predominant losses of genetic material in a variety of chromosomal regions, including the whole chromosomes 10, 16, 17, 21, and 22 and numerous loci containing the principal tumour suppressor genes (*TP53*, *NF1*, *PTEN*, *MGMT*, *CDKN2A*, etc.). Simultaneously, chromosomal regions containing protooncogenes associated with angiogenic and proliferative signalling pathways (*EGFR*, *MET*, *PDGFRA*, *CDK6*, and *BRAF*) have been amplified.

The large number of GO terms enriched, for both the deleted and amplified genes, highlights the impact of these alterations in critical processes such as DNA repair, damage response, cell cycle regulation, and chromatin remodelling.

In agreement with the favourable clinical outcome of this patient, we can highlight the loss and partial methylation of *MGMT* and loss of *RAD51* that promote a better response to TMZ, and *CFTR* suppresses GB cell proliferation associated with a longer progression-free survival. The *BRAF* alterations present in the reported patient are associated with better survival in a variety of tumours. It is also important to note that this patient exhibited genetic alterations that have been described as being associated with a poorer prognosis and reduced survival. In the future, studies with larger cohorts are necessary to understand the significance of these genetic alterations as a whole and their true impact on treatment response and survival in GB patients.

The monitoring of cfDNA levels revealed changes that may closely represent the clinical course of the disease. An initial rise in cfDNA concentration following the start of the STUPP protocol (QT-RT) likely reflected tumour cell apoptosis induced by treatment, and the subsequent decline suggested a favourable therapeutic response. Notably, an increase in cfDNA levels preceded imaging-confirmed tumour progression and the second surgical intervention, indicating that cfDNA may serve as an early and accessible biomarker for disease recurrence. These findings seem to support the integration of liquid biopsy approaches into clinical practice to enhance the management of GB patients, translating the possibility of correlating tumour resistance/sensitivity profiles, detection of minimal residual disease, anticipation of clinical/imaging progression, monitoring, and adaptation of highly personalised and individualised medical–surgical treatments, which are likely to improve the clinical guidance of these deleterious brain tumours.

According to the clinical records, the patient consistently stated that his will to continue working was the driving force behind the cure of the disease.

## Data Availability

The data presented in the study are deposited in the https://apps.uc.pt/mypage/faculty/fcaramelo/en/glio.
